# Conserved genes and pathways in primary human fibroblast strains undergoing replicative and radiation induced senescence

**DOI:** 10.1186/s40659-016-0095-2

**Published:** 2016-07-28

**Authors:** Shiva Marthandan, Uwe Menzel, Steffen Priebe, Marco Groth, Reinhard Guthke, Matthias Platzer, Peter Hemmerich, Christoph Kaether, Stephan Diekmann

**Affiliations:** 1 Leibniz Institute for Age Research-Fritz Lipmann Institute e.V. (FLI), Beutenbergstrasse 11, 07745 Jena, Germany; 2 Leibniz Institute for Natural Product Research and Infection Biology-Hans-Knöll-Institute e.V. (HKI), Jena, Germany

**Keywords:** Senescence, Fibroblasts, γ-irradiation, Aging, Transcriptome analysis, DNA repair

## Abstract

**Background:**

Cellular senescence is induced either internally, for example by replication exhaustion and cell division, or externally, for example by irradiation. In both cases, cellular damages accumulate which, if not successfully repaired, can result in senescence induction. Recently, we determined the transcriptional changes combined with the transition into replicative senescence in primary human fibroblast strains. Here, by γ-irradiation we induced premature cellular senescence in the fibroblast cell strains (HFF and MRC-5) and determined the corresponding transcriptional changes by high-throughput RNA sequencing.

**Results:**

Comparing the transcriptomes, we found a high degree of similarity in differential gene expression in replicative as well as in irradiation induced senescence for both cell strains suggesting, in each cell strain, a common cellular response to error accumulation. On the functional pathway level, “Cell cycle” was the only pathway commonly down-regulated in replicative and irradiation-induced senescence in both fibroblast strains, confirming the tight link between DNA repair and cell cycle regulation. However, “DNA repair” and “replication” pathways were down-regulated more strongly in fibroblasts undergoing replicative exhaustion. We also retrieved genes and pathways in each of the cell strains specific for irradiation induced senescence.

**Conclusion:**

We found the pathways associated with “DNA repair” and “replication” less stringently regulated in irradiation induced compared to replicative senescence. The strong regulation of these pathways in replicative senescence highlights the importance of replication errors for its induction.

**Electronic supplementary material:**

The online version of this article (doi:10.1186/s40659-016-0095-2) contains supplementary material, which is available to authorized users.

## Background

DNA is the repository of genetic information in each living cell, its integrity and stability is essential to life. DNA, however, is not inert; rather, it is subject to assault from cell-internal and environmental processes. Any resulting damage, if not repaired, will lead to mutation and possibly disease.

Cell-internally, DNA is subject to oxidative damage from metabolic byproducts such as free radicals. In addition, DNA replication is prone to error. The rate at which DNA polymerase incorporates incorrect nucleotides into newly synthesized DNA drives spontaneous mutations in an organism. While polymerase proofreading normally recognizes and corrects many of these errors, some 10^−4^ to 10^−6^ mutations per gamete for a given gene survive this process. DNA damage is also induced by the cellular environment, for example by UV-light and radiation of the cell [1]. An individual cell can suffer up to 10^6^ DNA changes per day.

Cells have evolved a number of mechanisms to detect and repair the various types of DNA damage, no matter whether this damage is caused by the environment or by errors in replication and cell division. If the rate of DNA damage exceeds the capacity of the cell to repair it, the accumulation of errors can overwhelm the cell [2–11] and lead to mutations and potentially to cancer. After major damage, the cell induces self-destruction by necrosis or apoptosis [12–14]. As a functional alternative to apoptosis, less damaged or replicatively exhausted but functional cells become senescent (“Hayflick limit” [15, 16]), an irreversible cell cycle arrested state experienced by all mitotically competent cells. It results from an intrinsic natural barrier to unlimited cell division exhibited by all normal somatic cells, including fibroblast [17–20]. Several mechanisms and pathways, especially the p53–p21 and p16–pRB pathways and telomere processing are involved in cellular senescence induction [15, 21–37].

The induction of apoptosis and senescence is considered to be part of a cellular cancer protection strategy [38]. Cellular senescence arrests the growth of cells at risk for malignant transformation in culture and in vivo [39–46], in this way preventing the spread of damage to the next cell generation [47]. Senescent cells accumulate over the life span of rodents and primates [48] and are found primarily in renewable tissues and in tissues that experience prolonged inflammation. Senescence-associated changes in gene expression are specific and mostly conserved within individual cell types [49]. Most differences between the molecular signatures of pre-senescent and senescent cells entail cell-cycle and metabolism-related genes [49], as well as genes encoding the secretory proteins that constitute the senescence associated secretory phenotype (SASP) [50–52].

Both, the accumulation of errors internally by replication and cell division (a slow process involving changes in telomeric processing) or externally by irradiation (comparably fast, not involving telomere shortening) can induce cellular senescence of practically indistinguishable phenotypes [53]. We therefore speculated that in both cases, the transition into senescence may correlate with the differential regulation of similar genes. Human fibroblasts are a well-established model for the investigation of cellular senescence [5, 54–56]. Recently, we determined the transcriptional changes associated with the transition into replicative senescence [49]. Here, by γ-irradiation we induced premature (accelerated) cellular senescence [51] in primary human fibroblast cell strains (HFF and MRC-5), determined the corresponding transcriptional changes by high-throughput RNA sequencing and compared the results with those for replicative senescence. Indeed, for both cell strains we found a high degree of similarity in differential gene expression in replicative as well as in irradiation induced senescence. However, we also identified that the senescence induction process imprints specific differences in the two transcriptomes.

## Methods

### Cell strains

Primary human MRC-5 fibroblasts (14 weeks gestation male, fibroblasts from normal lung, normal diploid karyotype) were obtained from ATCC (LGC Standards GmbH, Wesel, Germany). HFF (primary cells, *Homo sapiens*, fibroblasts from foreskin, normal diploid karyotype) cells were a kind gift of T. Stamminger (University of Erlangen, [57]).

### Cell culture

Cells were cultured as recommended by ATCC in Dulbeccos modified Eagles low glucose medium (DMEM) with l-glutamine (PAA Laboratories, Pasching, Austria), supplemented with 10 % fetal bovine serum (FBS) (PAA Laboratories). Cells were grown under 20 % O_2_ levels in a 9.5 % CO_2_ atmosphere at 37 °C. For sub-culturing, the remaining medium was discarded and cells were washed in 1× PBS (pH 7.4) (PAA Laboratories) and detached using trypsin/EDTA (PAA Laboratories). Primary fibroblasts were sub-cultured in a 1:4 [=2 population doublings (PDs)] or 1:2 (=1 PD) ratio. For stock purposes, cryo-conservation of the cell strains at various PDs were undertaken in cryo-conserving medium (DMEM + 10 % FBS + 5 % DMSO). Cells were immediately frozen at −80 °C and stored for 2–3 days. Afterwards, cells were transferred to liquid nitrogen for long time storage. Re-freezing and re-thawing was not performed to avoid premature senescence [58].

One vial of each of the two fibroblast cell strains (MRC-5 and HFF) were obtained and maintained in culture from an early PD. After obtaining enough stock on confluent growth of the fibroblasts in 75 cm^2^ flasks, cells were sub-cultured into three separate 75 cm^2^ flasks (“triplicates”) and were passaged until they were senescent in culture. We analyzed “technical” replicates in order to determine the experimental error of our technical approach. When using three samples from independent stocks (“biological” replicates), these might already differ in their transcriptome and/or proteome when starting our analysis, making it difficult to estimate the error of our experimental procedure.

### Induction of cellular senescence

Cellular senescence was induced by γ-irradiation. Human fibroblast strains were irradiated by ionizing radiation in a Gamma cell GC40 (MDS Nordion, Ottawa, Canada) using the radioactive isotope ^137^Cs as source. Exposure time was determined by correcting the irradiation dose of 1.23 Gy/min with the decay factor time equating to roughly 62 s/Gy. Young PD fibroblast strains (MRC-5 at PD 32, HFF at PD 16) were seeded 48 h before radiation exposure. Once cells were 70 % confluent, they were subjected to different doses of γ-irradiation (0, 2, 15, 20 Gy) at room temperature (RT) and subsequently cultured at 37 °C.

### Detection of SA-β galactosidase activity

The SA-β Gal assay was performed as described by [48] in either of the fibroblast strains at different time spans (after 0, 24, 48, 72, 96 and 120 h) after subjecting them to different doses of γ-irradiation (0, 2, 15, 20 Gy). Cells were washed in 1× PBS (pH 7.4) and fixed in 4 % paraformaldehyde (pH 7.4), 10 min at RT. After washing the cells in 1× PBS (pH 7.4), staining solution consisting of 1 mg/ml X-Gal, 8 mM citric acid/sodium phosphate pH 6,0, 5 mM K_3_Fe(CN)_6_, 5 mM K_4_Fe(CN)_6_, 150 mM NaCl, 2 mM MgCl_2_, was added. The enzymatic reaction occurred without CO_2_ for 4–16 h at 37 °C. After incubation, cells were washed in 1× PBS (pH 7.4) and, in order to visualize cell nuclei, DNA and SAHFs, mounted with 4′-6-diamidine-2-phenyl indole (DAPI) containing prolong gold antifade reagent (Invitrogen, Carlsbad, USA). Paired two-sample type 2 Student’s t-tests, assuming equal variances, were applied to determine the statistical significance of the SA-β gal assay results.

### Immunoblotting

For immunoblotting, 10,000 cells/µl were used. Immunodetection was performed using 5 %-powdered milk in PBS-T (1× PBS, pH 7.4 and 1 % Tween 20) for blocking (Roth, Germany). The optimal concentration of all the primary antibodies was estimated in human fibroblasts. Primary antibodies, anti-p21 mouse antibody (OP64; Calbiochem; dilution 1:200), anti-p16 mouse antibody (550834; BD Pharmingen; 1:200), anti-IGFBP7 rabbit antibody (ab74169; Abcam; 1:500), anti-IGFBP5 rabbit antibody (ab4255; Abcam; 1:500), anti-IGFBP3 goat antibody (ab77635; Abcam; 1:500), anti-Id3 mouse antibody (ab55269; Abcam; 1:100), anti-BAX rabbit antibody (ab10813; Abcam; 1:200), anti-Caspase-3 rabbit antibody (ab2302; Abcam; 1:500) and anti-tubulin mouse antibody (T-9026; SIGMA-Aldrich; 1:5000) were diluted in 5 %-powdered milk (in PBS-T) and incubated for 1 h at RT. Washing steps were performed three times for 10 min in 1× PBS-T. The secondary horseradish peroxidase-labeled antibodies (Jackson Immuno Research Lab) were incubated for 1 h at RT. Horseradish peroxidase was detected using an ECL-detection system and radiographic film (GE Healthcare, Germany). After film development, signal intensities of immunoblot bands were quantified using Metamorph software [59]. The signal intensity values were examined for statistical significance using paired two-sample type 2 Student’s t-tests assuming equal variances.

### RNA extraction

Total RNA was isolated using Qiazol (Qiagen, Hilden, Germany) according to the manufacturer’s protocol, with modifications. In brief, the fibroblasts were pelleted in 2 ml safe-lock tubes (Eppendorf, Hamburg, Germany). 1 ml cooled Qiazol and one 5 mm stainless steel bead (Qiagen) were added. Homogenization was performed using a TissueLyzer II (Qiagen) at 20 Hz for 1 min. After incubation for 5 min at RT, 200 ml chloroform was added. The tube was shaken for 15 s and incubated for 3 min at RT. Phase separation was achieved by centrifugation at 12,000×*g* for 20 min at 4 °C. The aqueous phase was transferred into a fresh cup and 10 mg of glycogen (Invitrogen, Darmstadt, Germany), 0.16 volume NaOAc (2 M, pH 4.0) and 1.1 volume isopropanol were added, mixed and incubated for 10 min at RT. The RNA was precipitated by centrifugation with 12,000×*g* at 4 °C for 20 min. The supernatant was removed and the pellet was washed with 80 % ethanol twice and air dried for 10 min. The RNA was re-suspended in 20 μl DEPC-treated water by pipetting up and down, followed by incubation at 65 °C for 5 min. The RNA was quantified with a NanoDrop 1000 (PeqLab, Erlangen, Germany) and stored at −80 °C until use.

### RNA-seq

To ensure appropriate RNA quality and evaluate RNA degradation, total RNA was analyzed using Agilent Bioanalyzer 2100 (Agilent Technologies, USA) and RNA 6000 Nano Kit (Agilent). An average RNA integrity number (RIN) of 8 was obtained. Total RNA was used for Illumina library preparation and RNA-seq [60]. 2.5 µg total RNA was used for indexed library preparation using Illumina’s TruSeq™ RNA Sample Prep Kit v2 following the manufacturer’s instruction. Libraries were pooled and sequenced (five samples per lane) using a HiSeq 2000 (Illumina) in single read mode with 50 cycles using sequencing chemistry v3. Sequencing resulted in approximately 40 million reads with a length of 50 bp (base pairs) per sample. Reads were extracted in FastQ format using CASAVA v1.8.2 or v1.8.3 (Illumina).

### RNA-seq data analysis

Raw sequencing data were obtained in FASTQ format. Read mapping was performed using Tophat 2.0.6 [61] and the human genome references assembly GRCh37 (http://feb2012.archive.ensembl.org/). The resulting SAM alignment files were processed using the HTSeq Python framework and the respective GTF gene annotation, obtained from the Ensembl database [62]. Gene counts were further processed using the R programming language [63] and normalized to reads per kilobase of transcript per million mapped reads (RPKM) values. In order to examine the variance and the relationship of global gene expression across the samples, different correlation coefficients were computed including Spearman’s correlation of gene counts and Pearson’s correlation of log2 RPKM values.

Subsequently, the Bioconductor packages DESeq [64] and edgeR [65] were used to identify differentially expressed genes (DEG). Both packages provide statistics for determination of differential expression in digital gene expression data using a model based on the negative binomial distribution. Here we used non-normalized gene counts since both packages include internal normalization procedures. The resulting p values were adjusted using the Benjamini and Hochberg’s approach for controlling the false discovery rate (FDR) [66]. Genes with an adjusted p value <0.05 found by both packages were assigned as differentially expressed.

In our study, we applied DESeq [67, 68] instead of the recently presented alternative tool DESeq 2. DESeq 2 results in minor differences to DESeq, however showing a slightly lower median precision [69]. Applying the same statistical analysis tool (DESeq) for DEG identification allows a direct comparison of results in this study with those of our recent publications [35, 49, 70, 71].

### Sample clustering and analysis of variance

The variance and the relationship of global gene expression across the samples were examined by computing the Spearman correlation between all samples using genes with raw counts larger than zero. Furthermore, principal component analysis (PCA) was applied using the log2 RPKM values for genes with raw counts larger than zero.

### Gene set enrichment analysis to determine the most differentially regulated pathways on aging

We used the R package gage [72] in order to find significantly enriched Kyoto Encyclopedia of Genes and Genomes (KEGG) pathways. In case of our RNA-seq data, the calculation was based on the gene counts and was performed as described in the methods manual. Estimated p values were adjusted for multiple testing using the Benjamini and Hochberg’s approach for controlling false discovery rate. KEGG pathways were selected as significantly regulated if the corrected p values were smaller than 0.05.

## Results and discussion

Previously, changes in global gene expression have been studied during accelerated senescence induced by oncogenes in IMR-90 fibroblast strains [73, 74] or by chemotherapeutic drugs applied to tumor cells [37, 75] and during replicative and induced senescence in skin fibroblasts derived from Li-Fraumeni syndrome patients [76]. Here, we compared the transcriptomes of two γ-irradiation induced senescent human primary fibroblast strains with the corresponding transcriptomes of the replicatively senescent cells.

### Gamma irradiation resulted in senescence induction in primary human fibroblast strains

Mild irradiation (0.5 Gy) induces low levels of DNA damage in MRC-5 fibroblasts, followed by an increase of p21 protein levels [1, 51, 56]. After 3 days, the number of p21-positive cells drops to background levels, indicating successful DNA repair and return into the cell cycle. This mild irradiation did neither result in an increase of p16 protein levels nor in the up-regulation of the cellular senescence marker SA-β Gal [48]. After a slight time lag, the cell population continued to grow with the same rate as before, consistent with cell cycle re-entry after a transient cell cycle arrest [56]. After high dose irradiation (20 Gy), MRC-5 fibroblasts display a high number of repair foci which during the following days hardly decrease. After this high irradiation, not only p21 but now also p16 protein levels increase, associated with a complete cell proliferation arrest and a continuous increase of SA-β Gal positive cells [56]. Here, we subjected two different human fibroblast cell strains of different tissue origins [HFF (foreskin) and MRC-5 (embryonic lung)] to γ-irradiation, inducing premature cellular senescence. We determined the transcriptome of these irradiation-induced senescent cells in order to compare it with that of replicatively senescent cells of the same strains obtained by us before [49, 70].

**Fig. 1:**
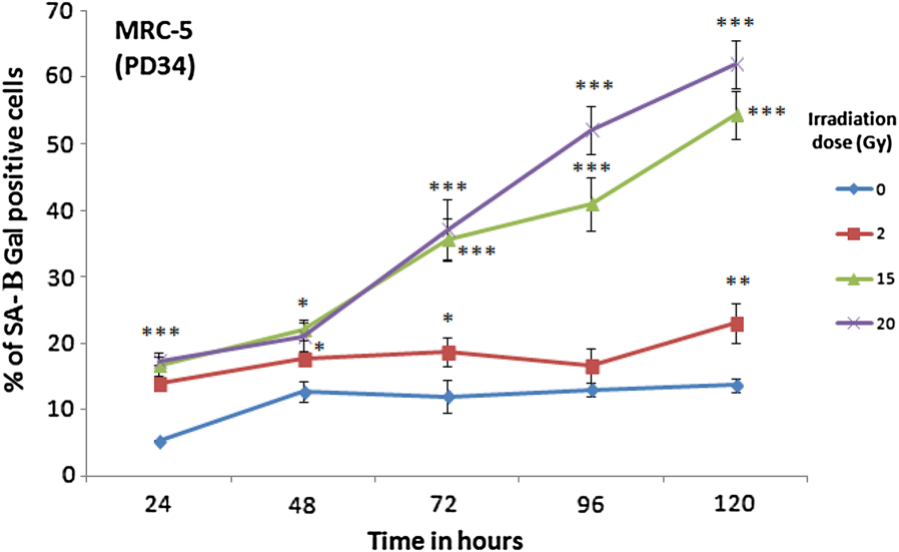
Percentage of SA-β gal positive cells in MRC-5 fibroblasts ±Gy irradiation. Young MRC-5 strains (PD34) were subjected to different doses of Gamma irradiation (0, 2, 15, 20 Gy) and the percentage of SA-β Gal positive cells were determined at different time points after irradiation treatment. Between 80 and 100 cells were analyzed for each data point. The* bars* indicate the mean ±SD. Values statistically different from their controls (0 Gy irradiation) are indicated with an *asterix* (t-test): *p < 0.05, **p < 0.01, ***p < 0.001. *n* = 3 in all cases

**Fig. 2:**
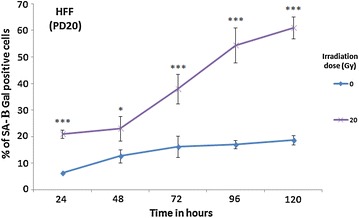
Percentage of SA-β Gal positive cells in HFF strains ± Gy irradiation. Young HFF strains (PD20) were subjected to no and 20 Gy of gamma irradiation (0, 20 Gy) and the percentage of SA-β gal positive cells were measured at different time points after irradiation treatment. Between 80 and 100 cells were analyzed for each data point. The *bars* indicate the mean ±S.D. Values statistically different from their controls (0 Gy irradiation) are indicated with an *asterix* (t-test): *p < 0.05, ***p < 0.001. *n* = 3 in all cases

MRC-5 fibroblasts were irradiated by 0, 2, 15, and 20 Gy at room temperature. Then, the percentage of SA-β Gal stained cells was determined at different time points over 5 days after irradiation treatment (Fig. 1). The highest percentage of SA-β Gal stained MRC-5 fibroblast cells (63 ± 4 %) was noted after the highest irradiation dose (20 Gy) and the longest time lapse (120 h) [72]. Therefore, HFF strains were only irradiated by 20 Gy. After 120 h the percentage of SA-β Gal stained HFF cells (62 ± 4 %; Fig. 2) was similar to the corresponding value for the MRC-5 fibroblasts. This time lapse, with the resulting degree of SA-β Gal staining, was selected since the transcriptomes of these cells will be compared to the corresponding transcriptomes of cells in replicative senescence of the same level of SA-β Gal staining (see below). 5 days after 20 Gy irradiation, MRC-5 cells are in early; partially still reversible but not yet in irreversible deep senescence [77]. At this time point (120 h after 20 Gy irradiation), immunoblotting revealed that increase of BAX expression [35, 78, 79] was not induced by 20 Gy irradiation. For caspase-3 [80], the levels of their active (cleaved, 17 kDa) form were not increased by 20 Gy irradiation. Since induction of BAX and cleavage of caspase-3 are more consistent with an induction of apoptosis, our results reveal that apoptosis was not induced (Additional file 1: Figure S1).This finding is consistent with earlier observations [1, 2, 81, 82]. Total MRC-5 and HFF sample RNA was extracted 120 h after 20 Gy irradiation and was subjected to RNA-seq.

### Transcriptome analysis of fibroblast strains subjected to irradiation induced senescence

**Fig. 3:**
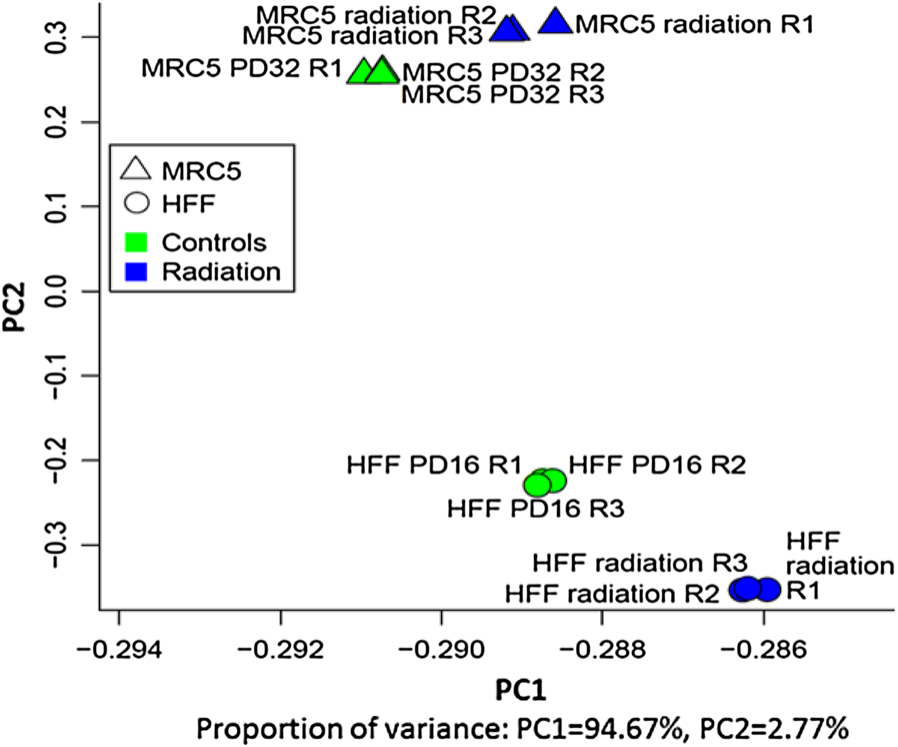
Variance and sample clustering of normalized transcriptome expression values principal component analysis (PCA) of young MRC-5 (*triangles*) and HFF (*spheres*) fibroblast cells of low PDs (MRC-5: 32, HFF: 16) subjected to 0 (control, *green*) and 20 Gy (*blue*) irradiation. Triplicates (*identical symbol and color*) are clustered indicating small experimental errors

Overall, the RNA-seq data were obtained from two samples, one for each cell strain (HFF and MRC-5), with three biological replicates each. The RNA-seq results revealed transcription of 27,410 and 27,944 genes for γ-irradiated HFF and MRC-5 fibroblasts, respectively. These were compared to the corresponding RNA-seq results of non-irradiated young (PD 16) HFF and young (PD 32) MRC-5 cells (obtained by us earlier [49, 70]). First, the RNA-seq retrieved normalized transcriptome expression values were analyzed using PCA. PCA reduces (by orthogonal transformation) high-dimensional data to 2 or 3 dimensions without losing much information, thereby enabling graphical visualization of the data. PCA is done in such a way that the first component of the graph shows as much of the variation contained in the data as possible. The PCA plot (Fig. 3) indicated a clear separation of the MRC-5 and HFF strains (PC2). The triplicates in all four cases clustered closely together, indicating small experimental errors (Fig. 3). The effect of irradiation induced senescence also exhibited similarities between the two fibroblast strains, demonstrated by the location and distance of both irradiated samples relative to the non-irradiated controls (irradiated samples on the right of controls; PC1).

In order to retrieve the most significant DEG, we applied stringent selection criteria of log2 fold change >1, p < 0.001 and adherence to both of the statistical packages edgeR and DESeq. 11,000 DEG in HFF and 6000 DEG in MRC-5 satisfied these selection criteria. An additional selection criterium of minimum RPKM >10 (in each of the samples compared; as mentioned in our previous studies [49, 70, 71]) resulted in more than 500 differentially regulated genes when comparing irradiated fibroblasts with their respective non-irradiated controls. Of these DEG, 29 % of the genes were commonly up- or down-regulated between HFF and MRC-5 fibroblasts (73 commonly up- and 70 commonly down-regulated). Thus, on the gene level HFF and MRC-5 cells respond only partially similar to irradiation, to a large extent the cellular response is cell strain specific. The heatmap comparison of the common most differentially regulated genes during both, replicative and irradiation induced senescence in both MRC-5 and HFF illustrates this point (Additional 2: Figure S2). In contrast, for their transition into replicative senescence we found a strong common gene regulation between HFF and MRC-5 [49] and among five human primary fibroblast strains (78 %) [70]. The strain specific response to irradiation is further supported by our observation that among the fifty most differentially regulated genes in MRC-5 and HFF strains, one commonly regulated gene was found, *TGFB2*. *TGFB2* is involved in the regulation of immune privilege, proliferation, differentiation and adhesion [83]. Furthermore, *TGFB2* is associated with senescence [84] and was found, as in these irradiated cells, significantly up-regulated in five replicatively senescent fibroblast strains including MRC-5 and HFF [70].

### Identical markers involved in replicative senescence and premature senescence induced by γ-irradiation

Recently, applying the same experimental procedure, we revealed the most significantly differentially expressed common genes during replicative senescence in HFF and MRC-5 fibroblasts [49]. As the next step, we compared these data with the irradiation results obtained here, by applying the stringency criteria of p < 0.001, and adherence to both statistical packages (edgeR and DESeq). For HFFs we found a total of 2589 commonly significantly differentially regulated genes in both, replication and irradiation induced senescent fibroblasts compared to controls. 2192 of these genes (85 %) were either up- or down-regulated in the same direction while the remaining 15 % were up-regulated in the one case but down in the other. Correspondingly, for MRC-5 we found a total of 936 commonly significantly differentially regulated genes in both, replication and irradiation induced senescent fibroblasts compared to controls. 689 of these genes (74 %) were either up- or down-regulated in the same direction. We thus found that for both fibroblast strains, the transition into replicative as well as irradiation induced senescence correlated with the common differential expression of a large number of genes and with a high degree of similarity in this common differential gene regulation. Interestingly, this common behaviour was observed for a considerably larger number of genes with a higher degree of similarity in HFF than in MRC-5. Our general conclusion is consistent with a recent study using human female lung diploid IMR-90 fibroblast strains [85]. Using Affymetrix arrays, this study compared RNA levels of 5 Gy γ-irradiation induced with replicatively senescent IMR-90 fibroblasts and found a number of genes differentially regulated in cells either arrested by irradiation or replicative exhaustion, with a strong overlap among regulated genes or showing a general trend in the same direction [85]. These data demonstrate the similarities in differential gene regulation between the two types of senescence induction and suggest that the majority of expression changes in replicatively senescent cells were due to proliferation arrest.

In HFF, among the most significant DEG in replication and irradiation-induced senescence were the genes *EGR1*, *Ki67*, *CCNB1*, *CCNA2*, *Id3*, *Id1*, *CLDN1*, *LIF*, *FBL,CST3*, *GRN* and *TMEM47*. Similarly, in MRC-5 these were *EGR1*, *Ki67*, *CCNB1*, *CCNA2*, *Id3*, *Id1, CLDN11, LIF*, *FBL*, *CTSK*, *MMP3* and *Wnt16* (stringency criteria: p < 0.001 and adherence to statistical packages, edgeR and DESeq). A number of these genes have cell cycle functions. GRN proteins play a role in wound healing [86]. Ki67 is a marker for proliferation [35, 87]. CTSK is normally stimulated by inflammatory cytokines released after tissue injury [88]. CST3 has been associated with aging-related loss of skeletal muscle (“sarcopenia”) [89]. Down-regulation of Id1 and Id3, as observed in our primary fibroblast strains, has been detected previously in BJ foreskin, WS1 fetal skin, and LF1 lung human fibroblasts [90]. Furthermore, Id loses function in cells transiting into senescence [91, 92]. CCNA2 is down-regulated in aging IMR-90 and WI-38 fibroblasts [93]. Expression of CCNB1 decreases due to antibiotic treatment, resulting in senescence induction in several cell types [94–96]. Reduced expression of CCNB1 inhibits the proliferation of breast cancer cells [97]. MMP3 up-regulation, as seen in our fibroblast strains, is reminiscent to their up-regulation during senescence in human melanocytes [98, 99]. Wnt16 is associated with senescence [100]. Thus, these genes are associated with proliferation, cell cycle arrest or senescence. We found here that these genes commonly correlate with senescence, independent of being irradiation induced or due to replicative exhaustion. Potentially, they are functionally involved in induction of senescence. As found for IL-6 and IL-8 [50, 51], we observed a significant increase in secretion of IL-11 in the media of HFF and MRC-5 fibroblasts undergoing replicative senescence compared to young control fibroblasts (data not shown). Here, we found the mRNA expression levels of the senescence associated secretory phenotype (SASP) family members *GRN*, *CTSK*, *CST3*, *MMP3* and *IGFBP7/5/3* up-regulated in irradiation induced senescent fibroblasts. These results are consistent with the establishment of a SASP [50].

Several of the above genes (*Ki67*, *CCNB1*, *CCNA2*, *LIF*, *FBL*, *CLDN1*, *WNT16*, *IGFBP3* and *IGFBP7*) were also among the commonly significantly differentially regulated genes during replicative and irradiation induced senescence in IMR-90 fibroblasts [85]. However, some of the significantly differentially regulated genes retrieved in [85] were not identified in our study. This difference could be attributed to the difference in (1) the fibroblast strain (IMR-90 strain [85] compared to HFF and MRC-5 in our study), (2) the technique used to retrieve the differentially expressed genes (Affymetrix arrays compared to RNA-seq in this study), (3) differences in stringency criteria of p < 0.05 and fold change >2 in [85] compared to p < 0.001 and adherence to both statistical packages (edgeR and DESeq) in the present study, and finally (4) the intensity of the Gy irradiation (5 Gy [85] compared to 20 Gy in our study).

### Impact of irradiation induced senescence on major transcription factors involved in cell survival

The transcription factors FOXM1 and E2F1 play an important role in cell survival [101–109]. As in both, MRC-5 and HFF fibroblast strains undergoing replicative senescence [70], *FOXM1* and *E2F1* were found here to be significantly (log2 fold change >1) down-regulated in irradiation induced senescent fibroblasts.

The down-regulation of *FOXM1* explains the significant down-regulation of the cell cycle associated genes *CENPF* and *CCNB2* [101, 102] in irradiation induced senescent cells. *FOXM1* has been revealed to have a positive feedback loop with Polo-like-kinase 1 (Plk1) and a negative feedback loop with p53 [110]. Furthermore, *FOXM1* has been functionally associated to the expression of X-ray cross-complementing group 1 (*XRCC1*) involved in base excision repair and breast cancer-associated gene 2 (*BRCA2*) dealing with homologous recombination repair of DNA double-strand breaks [111]. Similar to fibroblast strains undergoing replicative senescence [70], *Plk1* mRNA expression levels were significantly down-regulated, parallel to *FOXM1*, in irradiated fibroblasts whereas the expression levels of *p53*, *XRCC1* and *BRCA2* were not significantly different to controls.

E2F1 is associated with senescence and cell cycle function [112, 113]. Its downstream targets *p14*, *MMP1* and *MMP3* were found here not to be significantly differentially regulated in irradiation induced senescent cells. Expression levels of other transcription factors including *ATF1* [114, 115], *CREB1* [116], *NFκB1* [117] and *HSF1* [118, 119] revealed no significant differential expression on irradiation induced senescence. None of the five members of the NFκB family (*NFκB1*, *NFκB2*, *RelA*, *RelB*, *c*-*Rel*) were significantly differentially regulated in irradiation induced senescent cells. The lack of differential expression of E2F1, ATF1, CREB1, NFκB1 and HSF1 was also observed in senescence induced by replicative exhaustion. The significant differential regulation of FOXM1, E2F1, Plk1 and CENPF were also observed in the previous study [85] conducted in IMR-90 strains.

**Fig. 4:**
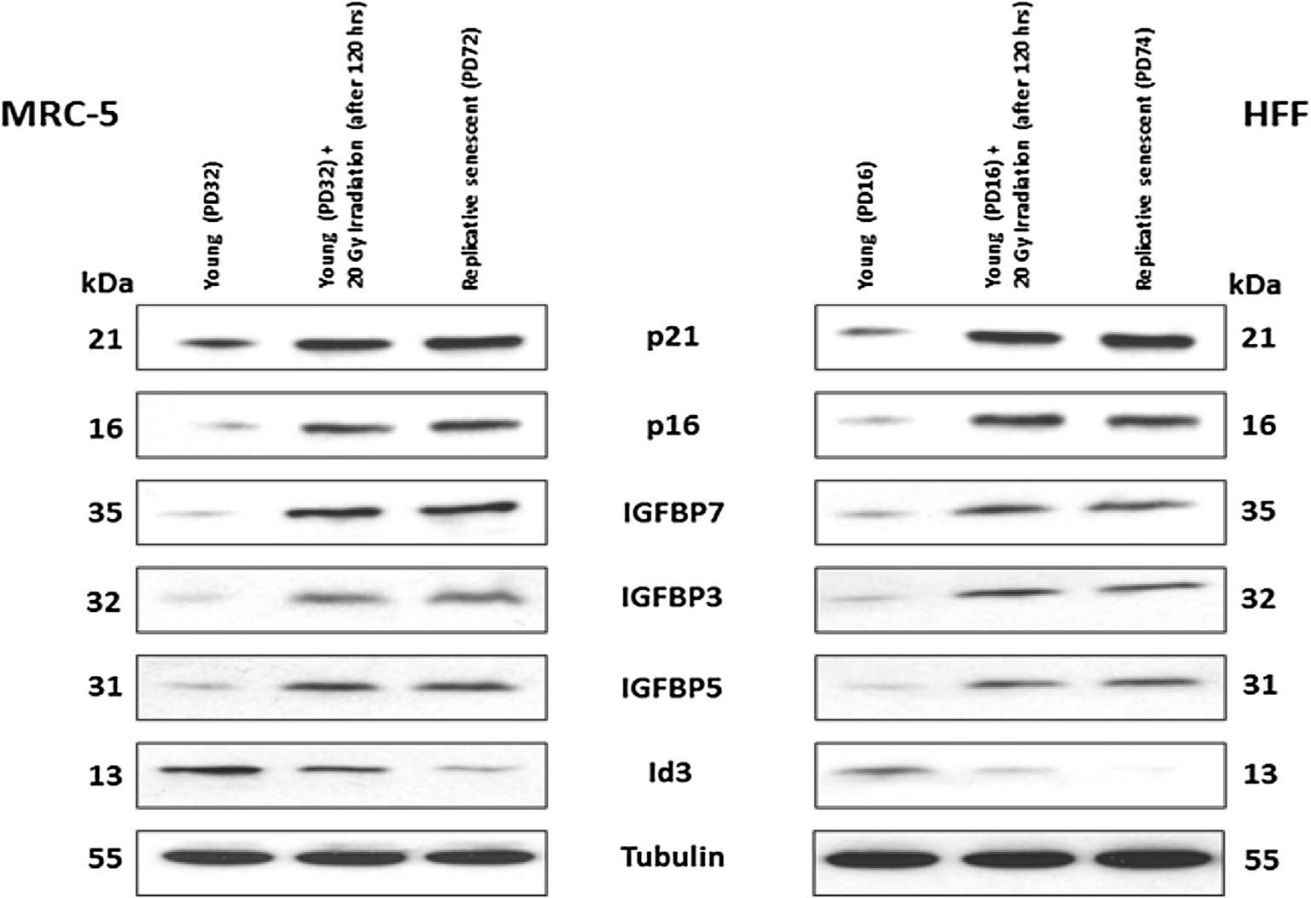
Immunoblots reveal protein expression levels of markers having a role in induction of senescence. The levels of induction of these proteins in fibroblasts at different cell states are demonstrated (low PD, low PD + 20 Gy irradiation (after 120 h), replicative senescence). The up- or down-regulation was signified by the presence or absence of the bands. p21, p16, IGFBP7, IGFBP3, IGFBP5 were up-regulated to a similar extent in irradiation induced as well as in replicatively senescent cells. In contrast, Id3 was down-regulated in both cases, in replicative stronger than in irradiation induced senescence, and in HFF stronger than in MRC-5 strains

**Fig. 5:**
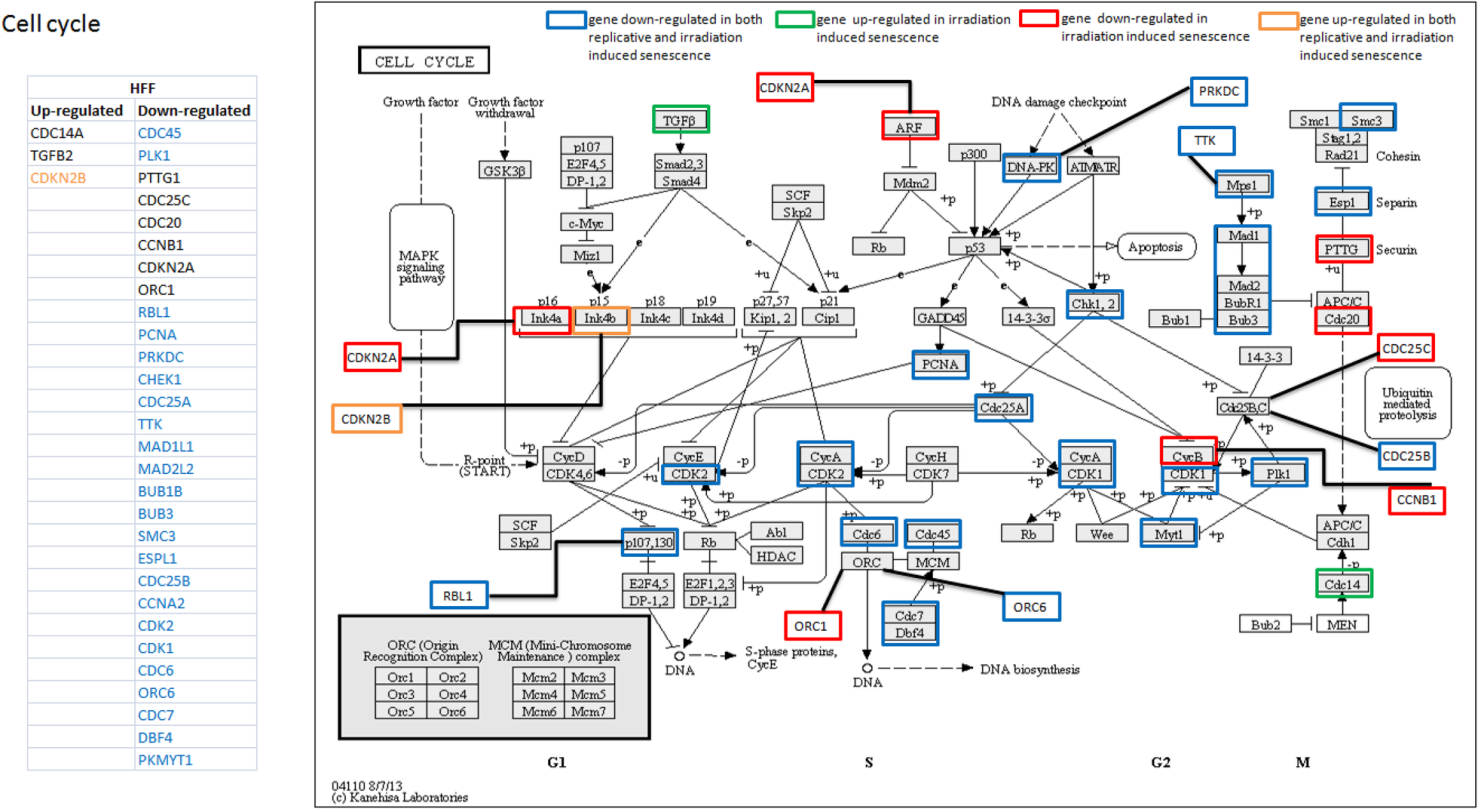
Regulation of genes of Cell Cycle pathway during senescence induction in HFF strains. Genes of the “cell cycle” pathway which are significantly up- (*green*) and down- (*red*) regulated (log2 fold change >1) during irradiation induced senescence (120 h after 20 Gy irradiation) in HFF strains. *Orange* and *blue* colors signify genes which are commonly up- (*orange*) and down-regulated (*blue*) during both, irradiation induced and replicative senescence

Interestingly, the mRNA expression levels of cyclin dependent kinase inhibitors (CDKIs) associated with senescence induction [4, 15, 27, 31, 120] were not among the significantly differentially regulated genes in irradiation induced senescent cells compared to controls in both, MRC-5 and HFF fibroblasts. However, protein expression levels of p21 and p16 were found to be significantly up-regulated in irradiation induced senescent fibroblasts compared to controls (Fig. 4). In fact, the mRNA expression level of CDKN2A (p16) was significantly down-regulated in HFF strains (Fig. 5). The selective lack of correlation of mRNA and protein expression levels has been observed before by [121–123] and by us in MRC-5 and HFF strains undergoing replicative senescence [70]. Thus, our results reveal that protein expression of p16 and p21 is regulated by other down-stream mechanisms than at transcriptional levels.

In contrast, protein expression levels of other selected markers associated with senescence in primary human fibroblast strains (Id3, IGFBP3, IGFBP5 and IGFBP7) revealed a good correlation with mRNA expression levels (Fig. 4). The mRNA and protein expression levels of all three IGFBP family members were significantly up-regulated in both HFF and MRC-5 senescent fibroblast strains, consistent with earlier observations [124–129]. A full proteomics analysis of the transition into senescence of human primary fibroblast strains is under way in our laboratory (to be published elsewhere).

### Retrieval of KEGG pathways significantly differentially regulated on irradiation induced senescence

Next, we retrieved the functional pathways significantly (p < 0.05) up- or down-regulated in irradiation induced senescent primary fibroblast strains.

After irradiation, we did not observe either an induction of BAX or a cleavage of caspase-3 (see above in “Gamma irradiation resulted in premature senescence induction in primary human fibroblast strains” section ), indicating that apoptosis was not induced. Analyzing the expression of genes involved in the “Apoptosis” KEGG pathway confirmed this finding: caspase gene family members and other genes having a role in apoptosis induction, including BAX, were not significantly up-regulated after irradiation in either of the two fibroblast strains.

**Fig. 6:**
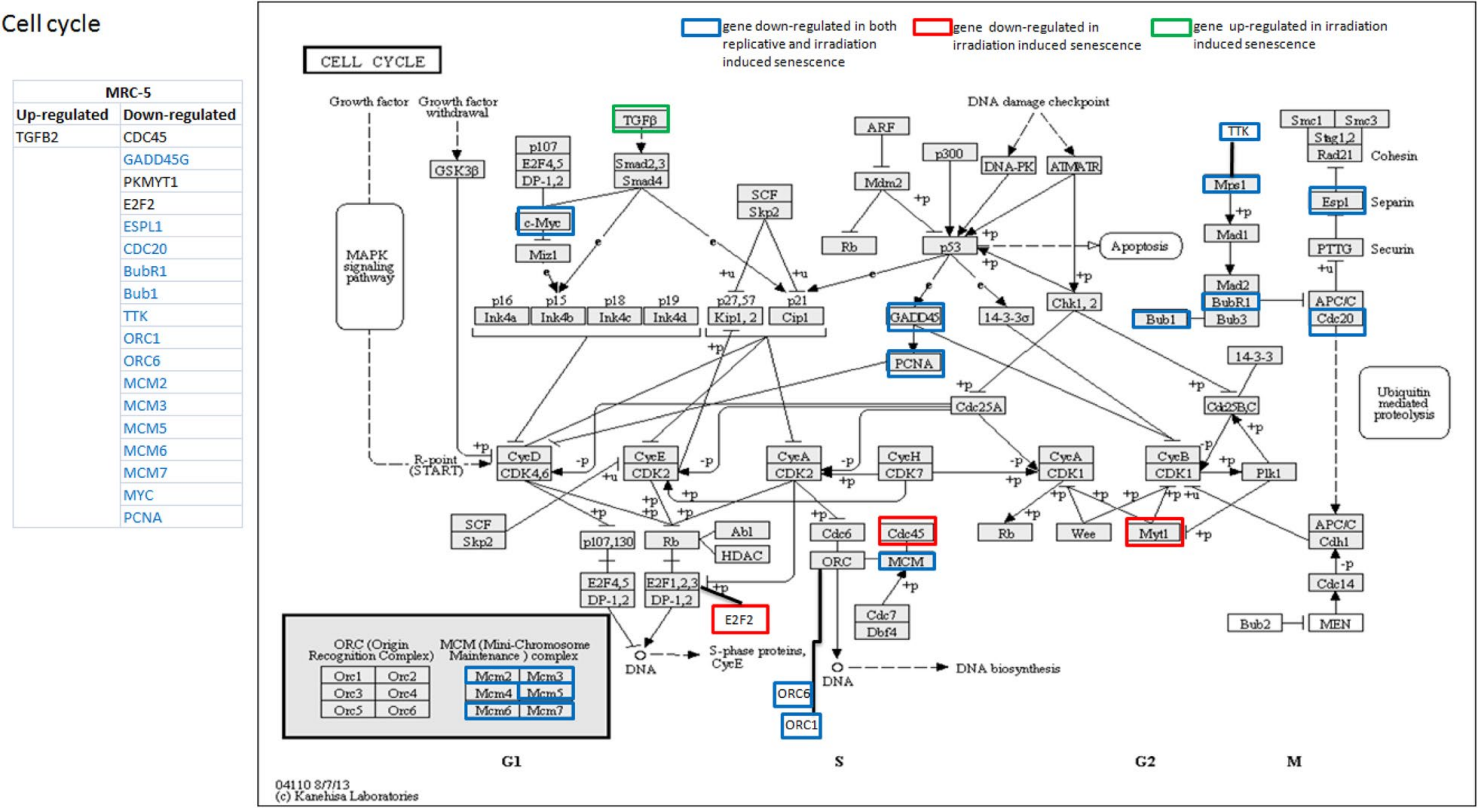
Regulation of genes of cell cycle pathway during senescence induction in MRC-5 strains. Genes of the “cell cycle” pathway which are significantly up- (*green*) and down- (*red*) regulated (log2 fold change >1) during irradiation induced senescence (120 h after 20 Gy irradiation) in MRC-5 fibroblasts. *Blue color* signifies genes which are commonly down-regulated during both, irradiation induced and replicative senescence

We compared irradiation induced pathways with those found in replicatively senescent cells of the same two fibroblast strains [49, 70]. In HFF strains, six KEGG pathways, namely “arrhythmogenic right ventricular cardiomyopathy”, “cell adhesion molecules”, “dilated cardiomyopathy”, “ECM receptor interaction”, “PPAR signaling pathway” and “long term depression”, were found significantly up-regulated during replicative as well as irradiation-induced senescence (Additional file 3: Figure S3, Additional file 4: Figure S4, Additional file 5: Figure S5, Additional file 6: Figure S6, Additional file 7: Figure S7, Additional file 8: Figure S8). Only the “cell cycle” pathway was commonly down-regulated during senescence induced by both means (Fig. 5). In MRC-5 fibroblast strains, “NOD like receptor signaling pathway”, “cell cycle” and “TGF Beta signaling pathway” were commonly down-regulated in both, irradiation-induced and replicative senescence (Fig. 6, Additional file 11: Figures S9, Additional file 12: S10). Thus, “cell cycle” was the only pathway similarly significantly differentially down-regulated across all four cases (in replicative and irradiation-induced senescence of both fibroblast strains). This finding is consistent with earlier results obtained for IMR-90 fibroblasts [85] and confirms the tight link of the control of DNA repair to cell cycle regulation.

SASPs are an important hallmark and functional mediator of senescent cells [50]. Unexpectedly, a number of cytokines and cytokine receptors (IL11, EGFR, CXCL-1,2,3,5,6,14) were significantly down-regulated on irradiation induced senescence in MRC-5 and HFF strains, resulting in a significant down-regulation of the KEGG pathway “cytokine–cytokine receptor interaction” (hsa04060) representing SASP. In contrast, TGFB2 was significantly up-regulated more than fivefold. Our results point to a heterogeneous regulation of SASP at the transcript level. Measuring protein levels directly, for example by antibody arrays [50], may provide a clearer picture of irradiation induced SASP regulation. Of further interest is the significant down-regulation of TGF-beta signaling pathway in both HFF and MRC-5 (Additional file 3: Figure S3, Additional file 9) strains.

The observed difference in significantly differentially regulated pathways on irradiation induced senescence between the two fibroblast strains is not due to experimental error since the strain triplicates cluster closely together (Fig. 3). The difference could be attributed to the strain differences in origin (MRC-5, embryonic lung; HFF, foreskin). Furthermore, their difference in PD numbers could also contribute to this difference: In our experiments, the MRC-5 cells (ordered from ATCC) had the starting PD 28 while we received HFF cells isolated from foreskin of primary human donors at PD 12.

While we found a strong similarity in the differential expression of genes for both senescence induction processes, we identified a difference on the level of functional pathways. Irradiation induced damage activates cellular repair processes [130, 131], often combined with p53, p21 and p16 mediated cell cycle arrest and, if repair is not successful, transition into senescence [1, 15, 132–135]. In irradiation induced senescence, only a few genes of the repair pathways were significantly down-regulated below control levels in MRC-5 fibroblasts (Additional file 10). In HFF strains we observed a down-regulation of all genes involved in the three DNA repair pathways, however, to a lesser extent than the down-regulation of other pathways. In contrast, in replicatively senescent cells all three repair pathways were significantly down-regulated in both fibroblast cell strains [49, 70]. Since we analyzed cells in early senescence, this quantitative difference could potentially indicate that replicatively senescent cells shut down repair pathways earlier than irradiation induced senescent cells during their transition into senescence.

Similarly, only a few genes of the “replication” pathway were found significantly down-regulated in irradiation induced senescent cells of both strains. Instead, “replication” was the pathway with nearly all genes significantly down-regulated in replicatively senescent fibroblast cells. Thus, during the transition into replicative senescence two essential functions, DNA repair and replication are more stringently regulated than during the transition into irradiation induced senescence. This is consistent with the view that replication errors are essential for the induction of replicative senescence while this process is not as relevant for irradiation induced senescence. Consequently, in replicative senescence the “replication” pathway is completely down-regulated.

Next, we analyzed the genes of significantly differentially regulated pathways (up- or down-regulated in either of the fibroblast strains) (Figs. 5, 6; Additional file 3: Figure S3, Additional file 4: Figure S4, Additional file 5: Figure S5, Additional file 6: Figure S6, Additional file 7: Figure S7, Additional file 8: Figure S8, Additional file 11: Figure S9, Additional file 12: Figure S10). We retrieved the expression levels of the involved genes (both up- and down-regulated) in the two fibroblast strains and identified which genes were commonly differentially regulated in both replicative and irradiation induced senescence (Figs. 5, 6; Additional file 3: Figure S3, Additional file 4: Figure S4, Additional file 5: Figure S5, Additional file 6: Figure S6, Additional file7: Figure S7, Additional file 8: Figure S8, Additional file 11: Figure S9, Additional file 12: Figure S10). The comparison with previous studies enabled us to functionally associate a number of these genes with induction of cell cycle arrest and senescence (highlighted in blue in Additional file 2). Several genes having a role in senescence induction (such as *TGFB2*, *IGF1*, *Id1*, *Id3*, *Id4*, *IL1B*, *IL6* and *IL8*) were among those genes (highlighted in blue in Additional file 10) which were similarly differentially regulated in both replicative and irradiation induced senescence [50, 55, 84, 90–92, 136–143]. Most importantly, the list (Additional file: 10) also includes on the one hand, genes which have not previously been associated with induction of senescence and, on the other hand, genes which are differentially regulated exclusively during irradiation induced senescence. In future studies, we intend to functionally validate the role of several of these genes in senescence induction by irradiation, replicative exhaustion or both.

## Conclusion

We compared the transcriptomes of two young and senescent human primary fibroblast strains, with the senescent state either induced by γ-irradiation or by replicative exhaustion. We found a strong similarity in the differential expression of genes for both senescence induction processes, indicating a considerably common cellular response to either internal or external damage. On the functional pathway level, “Cell cycle” was the only pathway commonly (down-) regulated in replicative and irradiation-induced senescence in both fibroblast strains, confirming the tight link between DNA repair and cell cycle regulation. In γ-irradiation induced senescence, only a few genes of the repair pathways were significantly down-regulated below control levels in MRC-5 strains. In HFF strains we observed a down-regulation of all genes involved in DNA repair pathways, however, to an extent less significant than the down-regulation of other pathways. In contrast, all three repair pathways are significantly down-regulated in replicatively senescent fibroblast cells. Furthermore, only a few genes of the “replication” pathway were found significantly down-regulated in irradiation induced senescent cells. Instead, “replication” was the pathway with nearly all genes significantly down-regulated in replicatively senescent fibroblasts. Thus, on the pathway level we identified considerable differences between both senescent states. During the transition into replicative senescence two essential functions, DNA repair and replication are more stringently regulated than during the transition into irradiation induced senescence, consistent with replication errors being essential for the induction of replicative senescence while this process is not as relevant for irradiation induced senescence.

## Additional files

10.1186/s40659-016-0095-2 Induction or lack of apoptotic induction in primary human fibroblast strains subjected to 20 Gy irradiation. The blots show the lack of induction of two different apoptotic markers (Caspase-3 or BAX) in primary human fibroblast strains (HFF and MRC-5), 120 min after 20 Gy irradiation. Induction of either of the apoptotic markers in non-irradiated early PD fibroblasts were used as controls. As a positive control, the fibroblasts were subjected to induction of apoptosis by treatment with 2.0 μM Stauroporine for 96 h [35, 205–208].
10.1186/s40659-016-0095-2 Heatmap showing the intersection of the most differentially expressed genes in each of the fibroblast strains (irradiated versus controls). Heatmap illustrating the log2 fold change of gene expression when comparing irradiated HFF versus controls (upper part) and irradiated MRC-5 versus controls (lower part) respectively. The horizontal axis displays the genes selected for this comparison. Genes were selected by intersecting the 200 most differentially regulated genes for each condition. This intersection contains 46 genes. The color key (top left) relates heatmap color to log2 fold change. Red color indicates a negative log2 fold change, i.e. a down-regulation under the second condition compared to the first condition, while the yellow color indicates a positive log2 fold change, i.e. an up-regulation under the second condition relative to the first condition. The dendro- gram on top of the plot clusters the genes into groups with similar expression levels for both comparisons. While most of the genes show similar log2 fold changes for both comparisons, some genes are up-regulated for one comparison, and down-regulated for the other comparison (CTGF, COLEC12, KRT19 in the middle of the plot).
10.1186/s40659-016-0095-2 Regulation of genes of Arrhythmogenic right ventricular cardiomyopathy pathway during senescence induction in HFF strains Genes of the “Arrhythmogenic right ventricular cardiomyopathy” pathway which are significantly up- (green) and down- (red) regulated (log2 fold change >1) during irradiation induced senescence (120 h after 20 Gy irradiation) in HFF strains. Orange color signifies genes which are commonly up-regulated during both, irradiation induced and replicative senescence.
10.1186/s40659-016-0095-2 Regulation of genes of Cell adhesion pathway during senescence induction in HFF strains. Genes of the “Cell adhesion” pathway which are significantly up- (green) and down- (red) regulated (log2 fold change >1) during irradiation induced senescence (120 h after 20 Gy irradiation) in HFF strains. Orange and blue colors signify genes which are commonly up- (orange) and down-regulated (blue) during both, irradiation induced and replicative senescence.
10.1186/s40659-016-0095-2 Regulation of genes of Dilated cardiomyopathy pathway during senescence induction in HFF strains. Genes of the “Dilated cardiomyopathy” pathway which are significantly up- (green) and down- (red) regulated (log2 fold change >1) during irradiation induced senescence (120 h after 20 Gy irradiation) in HFF strains. Orange color signifies genes which are commonly up-regulated during both, irradiation induced and replicative senescence.
10.1186/s40659-016-0095-2 Regulation of genes of ECM receptor interaction pathway during senescence induction in HFF strains. Genes of the “ECM receptor interaction” pathway which are significantly up- (green) and down- (red) regulated (log2 fold change >1) during irradiation induced senescence (120 h after 20 Gy irradiation) in HFF strains. Orange and blue colors signify genes which are commonly up- (orange) and down-regulated (blue) during both, irradiation induced and replicative senescence.
10.1186/s40659-016-0095-2 Regulation of genes of PPAR signaling pathway during senescence induction in HFF strains. Genes of the “PPAR signaling” pathway which are significantly up- (green) and down- (red) regulated (log2 fold change >1) during irradiation induced senescence (120 h after 20 Gy irradiation) in HFF strains. Orange and blue colors signify genes which are commonly up- (orange) and down-regulated (blue) during both, irradiation induced and replicative senescence.
10.1186/s40659-016-0095-2 Regulation of genes of Long term depression pathway during senescence induction in HFF strains. Genes of the “Long term depression” pathway which are significantly up- (green) and down- (red) regulated (log2 fold change >1) during irradiation induced senescence (120 h after 20 Gy irradiation) in HFF strains. Orange color signifies genes which are commonly up- regulated during both, irradiation induced and replicative senescence.
10.1186/s40659-016-0095-2 Regulation and function of genes associated with repair pathways during irradiation induced senescence. Differential regulation and function of (i) genes associated with DNA repair (Nucleotide excision, Base excision, Mismatch repair) and (ii) DNA replication pathways on 20 Gy irradiation induced senescence in MRC-5 fibroblasts and HFF strains. Blue color denotes that previous studies have functionally associated these genes with induction of senescence.
10.1186/s40659-016-0095-2 Function of genes associated with common differentially regulated pathways during replicative and pre-mature senescence induction. Function of each of the genes associated with the commonly differentially regulated pathways (up- and down-regulated) on replicative and 20 Gy irradiation induced senescence in the MRC-5 fibroblasts and HFF strains. Blue color denotes that previous studies have functionally associated these genes with induction of senescence.
10.1186/s40659-016-0095-2 Regulation of genes of NOD-like receptor signaling pathway during senescence induction in MRC-5 strains. Genes of the “NOD-like receptor signaling” pathway which are significantly down- (red) regulated (log2 fold change >1) during irradiation induced senescence (120 h after 20 Gy irradiation) in MRC-5 fibroblast strains. Blue color signifies genes which are commonly down-regulated during both, irradiation induced and replicative senescence.
10.1186/s40659-016-0095-2 Regulation of genes of TGF-beta signaling pathway during senescence induction in MRC-5 strains. Genes of the “TGF-beta signaling” pathway which are significantly up- (green) and down- (red) regulated (log2 fold change >1) during irradiation induced senescence (120 h after 20 Gy irradiation) in MRC-5 fibroblast strains. Blue color signifies genes which are commonly down-regulated during both, irradiation induced and replicative senescence.

## Abbreviations

HFF: human foreskin fibroblasts
DMEM: Dulbeccos modified Eagles low glucose medium
FBS: fetal bovine serum
CO_2_: carbondioxide
PD: population doubling
RT: room temperature
DAPI: 4′-6-diamidine-2-phenyl indole
PCA: principle component analysis
RPKM: reads per kilo base per million mapped reads
FDR: false discovery rate
SA β-Gal: senescence associated β-Gal
RNA-seq: high-throughput RNA sequencing
CDKI: cyclin dependent kinase inhibitors
GAGE: gene set enrichment for pathway analysis
KEGG: Kyoto encyclopedia of genes and genomes
UV: ultraviolet
pRB: phosphorylated retinoblastoma protein
PBS: phosphate buffered saline
RIN: RNA integrity number
DEG: differentially expressed genes
CTSK: cathepsin K
TMEM47: transmembrane protein 47
CCNB1: cyclin B1
CCNA2: cyclin A2
Wnt-16: protein Wnt-16
IGFBP3: insulin-like growth factor-binding protein 3
IGFBP5: insulin-like growth factor-binding protein 5
IGFBP7: insulin-like growth factor-binding protein 7
p16: cyclin-dependent kinase inhibitor 2A
MMPs: matrix metallopeptidase
FOXM1: forkhead Box M1
ATF1: activating transcription factor 1
CREB1: CAMP responsive element binding protein 1
HSF1: heat shock transcription factor 1
hrs: hours
NFκB1: NF kappa B signaling
TGFB2: transforming growth factor beta 2
EGR1: early growth response 1
CLDN: claudin
LIF: leukemia inhibitory factor
FBL: fibrillarin
CST3: cystatin C
Id: dNA binding protein inhibitor
NaOAc: sodium acetate
GRN: granulin
K_3_Fe(CN)_6_: potassium ferricyanide
K_4_Fe(CN)_6_: potassium ferrocyanide
EDTA: ethylenediaminetetraacetic acid
DMSO: dimethyl sulfoxide
FOXM1: forkhead box protein M1
E2F1: transcription factor E2F1
PlK1: polo-like-kinase 1
XRCC1: X-ray repair cross-complementing protein 1
BRCA2: breast cancer type 2 susceptibility protein
ECM: extracellular matrix
PPAR: peroxisome proliferator-activated receptors
NOD: nucleotide-binding oligomerization domain receptors
IL: interleukin
IL1B: interleukin 1 beta
