# Supplementary figures and images for: Conserved genes and pathways in primary human fibroblast strains undergoing replicative and radiation induced senescence
Source: Biol Res. 2016 Jul 28;49:34. doi: 10.1186/s40659-016-0095-2 (PMC4963952; doi:10.1186/s40659-016-0095-2)

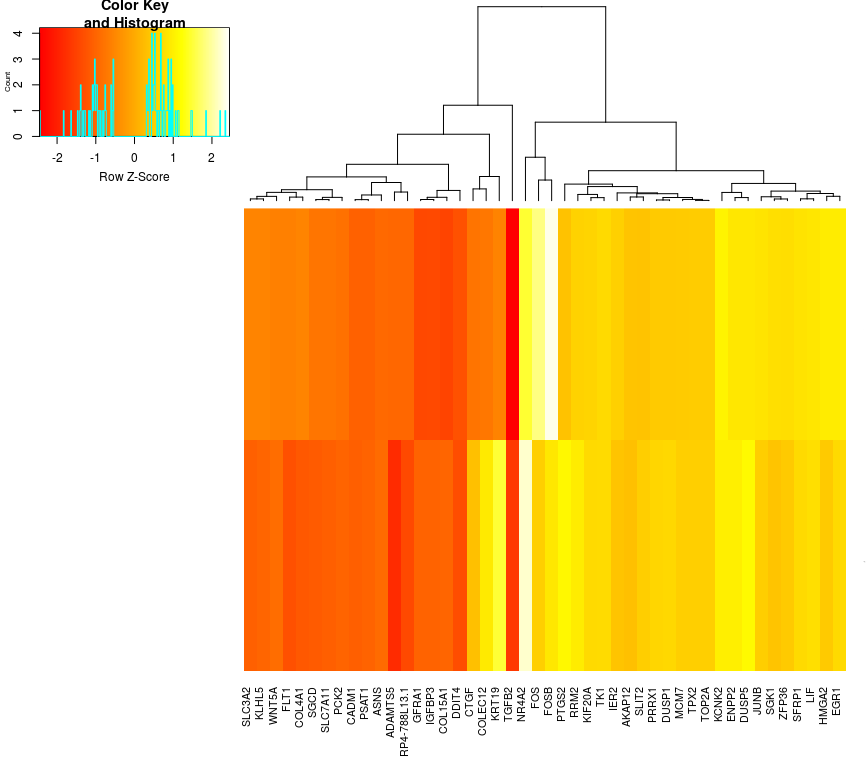

Supplement: Supplementary file 2 — 10.1186/s40659-016-0095-2 Heatmap showing the intersection of the most differentially expressed genes in each of the fibroblast strains (irradiated versus controls). Heatmap illustrating the log2 fold change of gene expression when comparing irradiated HFF versus controls (upper part) and irradiated MRC-5 versus controls (lower part) respectively. The horizontal axis displays the genes selected for this comparison. Genes were selected by intersecting the 200 most differentially regulated genes for each condition. This intersection contains 46 genes. The color key (top left) relates heatmap color to log2 fold change. Red color indicates a negative log2 fold change, i.e. a down-regulation under the second condition compared to the first condition, while the yellow color indicates a positive log2 fold change, i.e. an up-regulation under the second condition relative to the first condition. The dendro- gram on top of the plot clusters the genes into groups with similar expression levels for both comparisons. While most of the genes show similar log2 fold changes for both comparisons, some genes are up-regulated for one comparison, and down-regulated for the other comparison (CTGF, COLEC12, KRT19 in the middle of the plot). [file 40659_2016_95_MOESM2_ESM.docx]

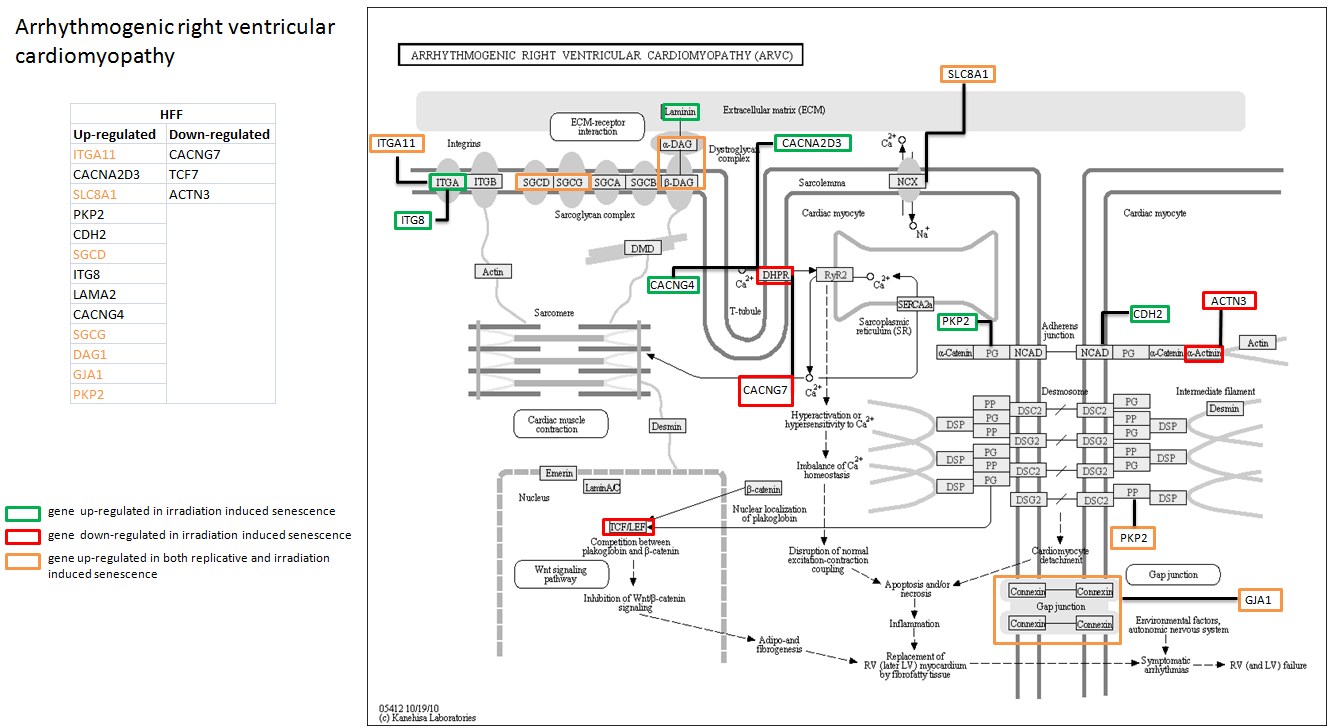

Supplement: Supplementary file 3 — 10.1186/s40659-016-0095-2 Regulation of genes of Arrhythmogenic right ventricular cardiomyopathy pathway during senescence induction in HFF strains Genes of the “Arrhythmogenic right ventricular cardiomyopathy” pathway which are significantly up- (green) and down- (red) regulated (log2 fold change >1) during irradiation induced senescence (120 h after 20 Gy irradiation) in HFF strains. Orange color signifies genes which are commonly up-regulated during both, irradiation induced and replicative senescence. [file 40659_2016_95_MOESM3_ESM.docx]

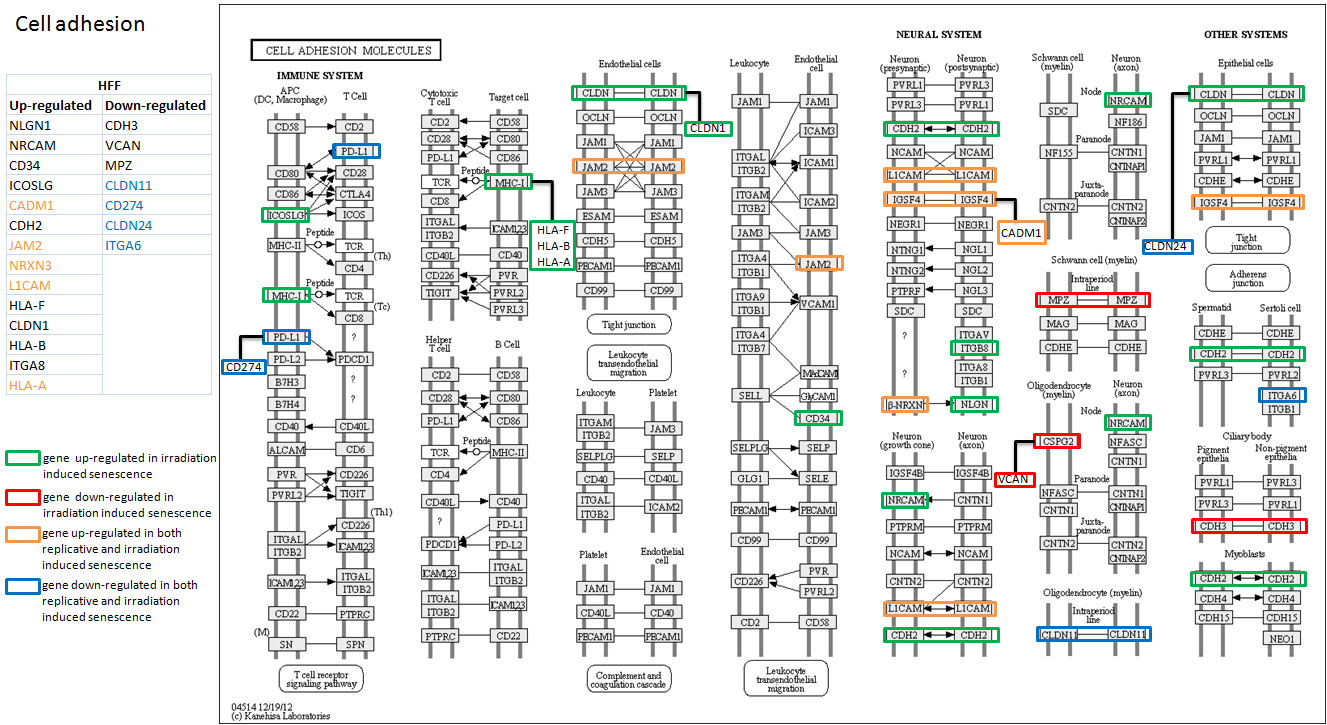

Supplement: Supplementary file 4 — 10.1186/s40659-016-0095-2 Regulation of genes of Cell adhesion pathway during senescence induction in HFF strains. Genes of the “Cell adhesion” pathway which are significantly up- (green) and down- (red) regulated (log2 fold change >1) during irradiation induced senescence (120 h after 20 Gy irradiation) in HFF strains. Orange and blue colors signify genes which are commonly up- (orange) and down-regulated (blue) during both, irradiation induced and replicative senescence. [file 40659_2016_95_MOESM4_ESM.docx]

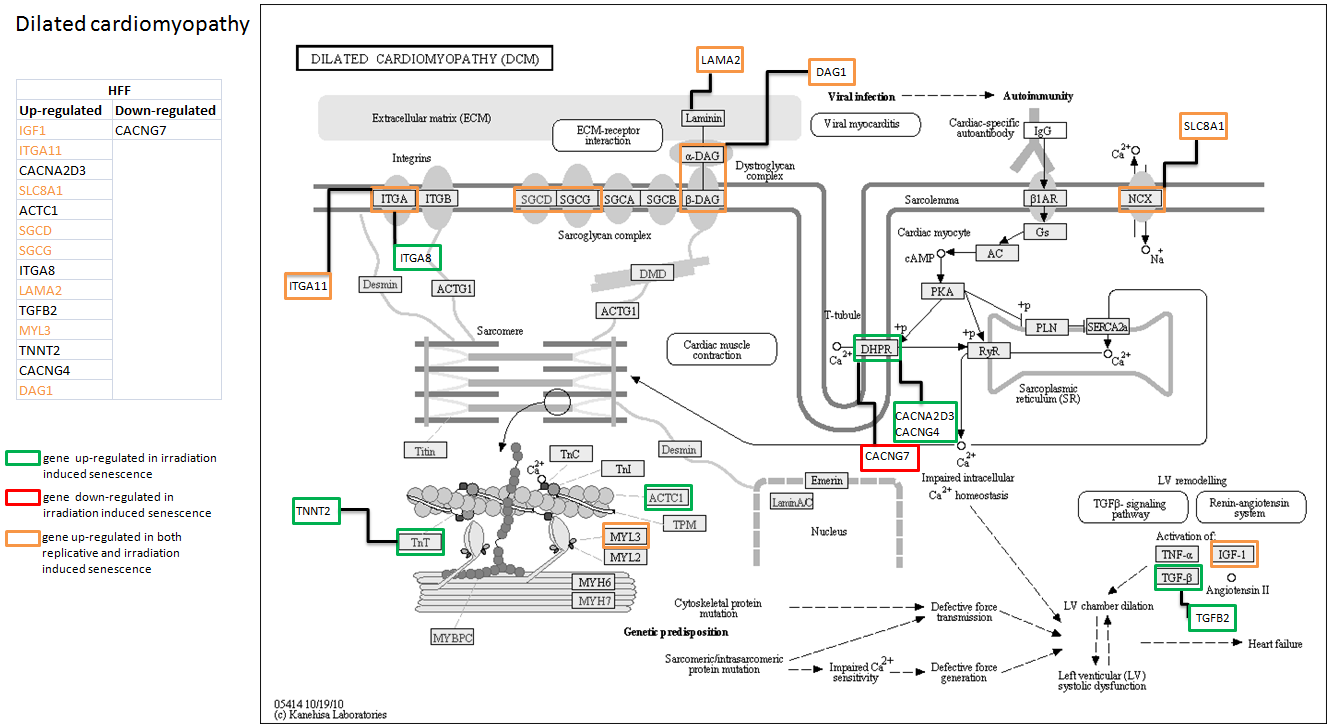

Supplement: Supplementary file 5 — 10.1186/s40659-016-0095-2 Regulation of genes of Dilated cardiomyopathy pathway during senescence induction in HFF strains. Genes of the “Dilated cardiomyopathy” pathway which are significantly up- (green) and down- (red) regulated (log2 fold change >1) during irradiation induced senescence (120 h after 20 Gy irradiation) in HFF strains. Orange color signifies genes which are commonly up-regulated during both, irradiation induced and replicative senescence. [file 40659_2016_95_MOESM5_ESM.docx]

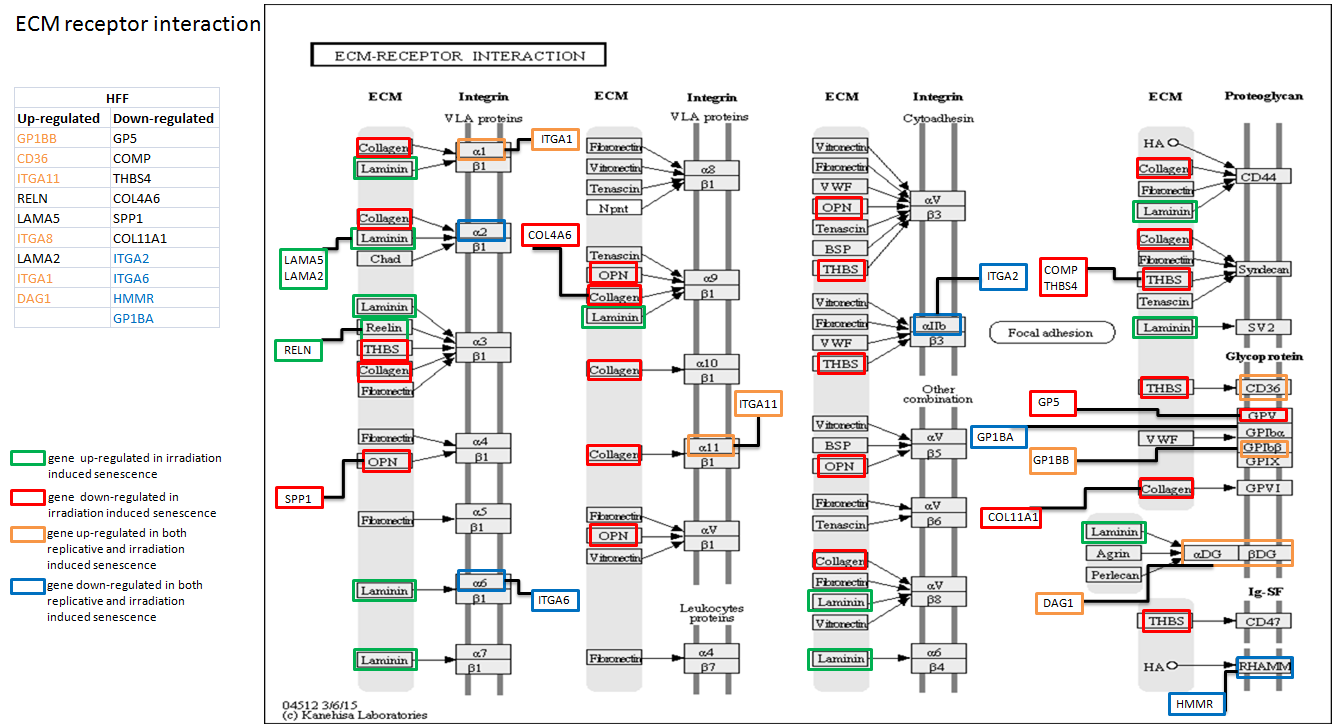

Supplement: Supplementary file 6 — 10.1186/s40659-016-0095-2 Regulation of genes of ECM receptor interaction pathway during senescence induction in HFF strains. Genes of the “ECM receptor interaction” pathway which are significantly up- (green) and down- (red) regulated (log2 fold change >1) during irradiation induced senescence (120 h after 20 Gy irradiation) in HFF strains. Orange and blue colors signify genes which are commonly up- (orange) and down-regulated (blue) during both, irradiation induced and replicative senescence. [file 40659_2016_95_MOESM6_ESM.docx]

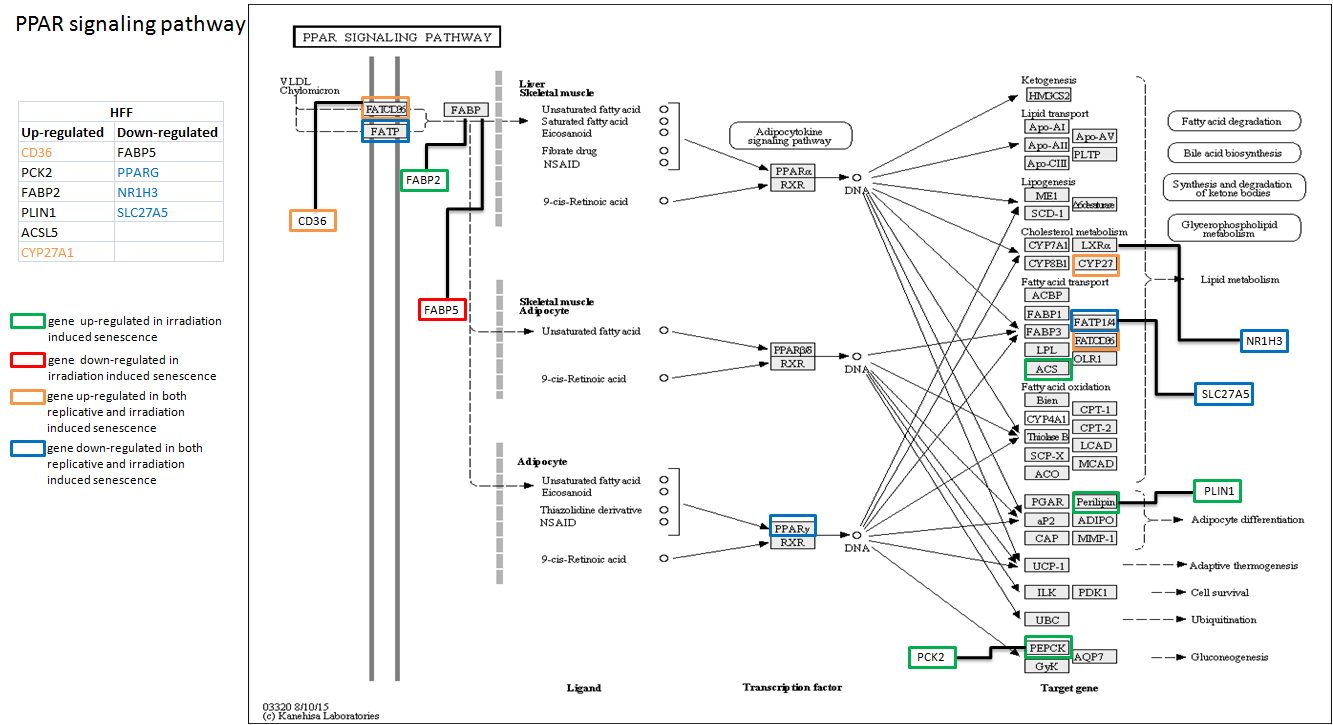

Supplement: Supplementary file 7 — 10.1186/s40659-016-0095-2 Regulation of genes of PPAR signaling pathway during senescence induction in HFF strains. Genes of the “PPAR signaling” pathway which are significantly up- (green) and down- (red) regulated (log2 fold change >1) during irradiation induced senescence (120 h after 20 Gy irradiation) in HFF strains. Orange and blue colors signify genes which are commonly up- (orange) and down-regulated (blue) during both, irradiation induced and replicative senescence. [file 40659_2016_95_MOESM7_ESM.docx]

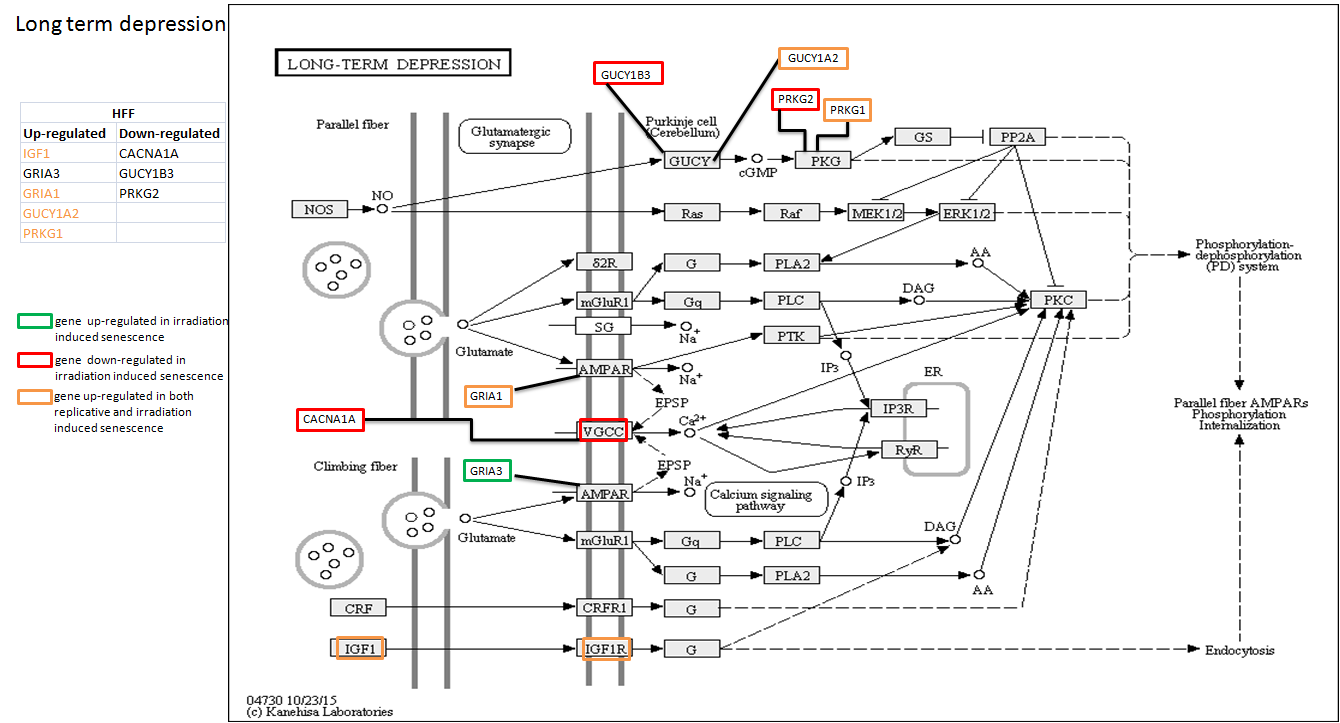

Supplement: Supplementary file 8 — 10.1186/s40659-016-0095-2 Regulation of genes of Long term depression pathway during senescence induction in HFF strains. Genes of the “Long term depression” pathway which are significantly up- (green) and down- (red) regulated (log2 fold change >1) during irradiation induced senescence (120 h after 20 Gy irradiation) in HFF strains. Orange color signifies genes which are commonly up- regulated during both, irradiation induced and replicative senescence. [file 40659_2016_95_MOESM8_ESM.docx]

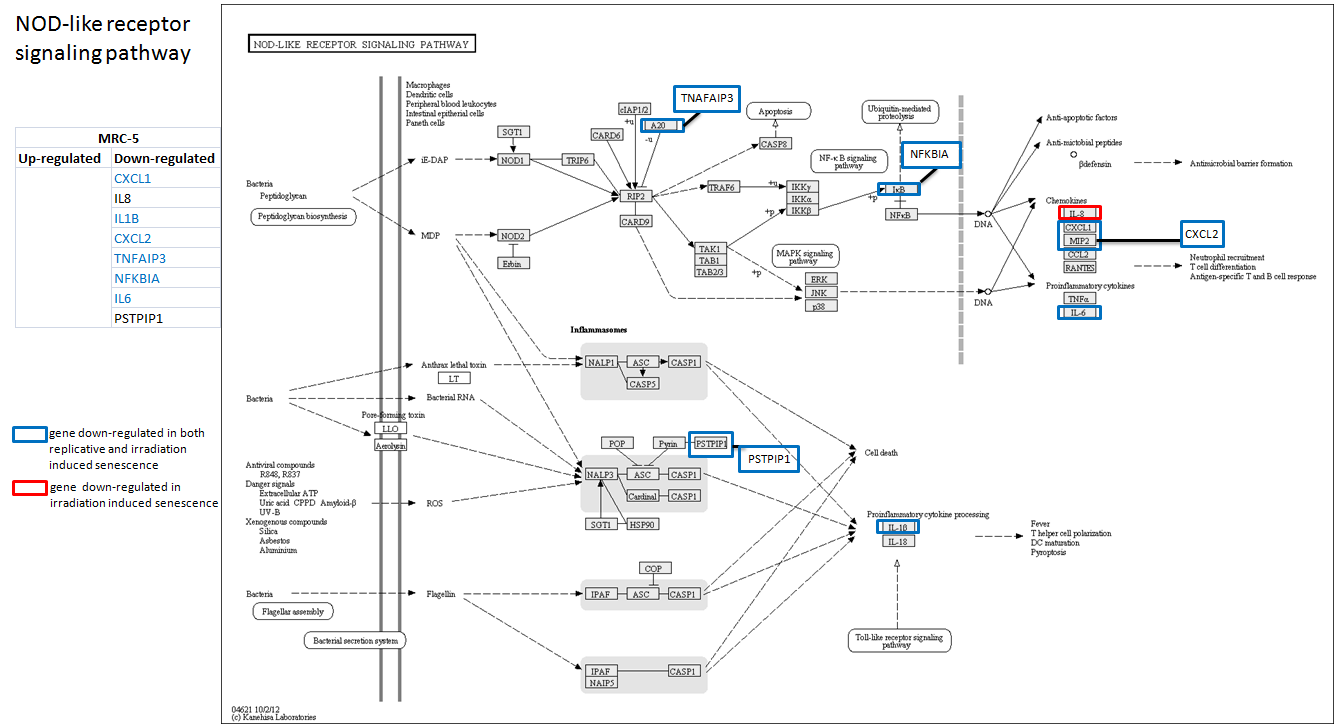

Supplement: Supplementary file 11 — 10.1186/s40659-016-0095-2 Regulation of genes of NOD-like receptor signaling pathway during senescence induction in MRC-5 strains. Genes of the “NOD-like receptor signaling” pathway which are significantly down- (red) regulated (log2 fold change >1) during irradiation induced senescence (120 h after 20 Gy irradiation) in MRC-5 fibroblast strains. Blue color signifies genes which are commonly down-regulated during both, irradiation induced and replicative senescence. [file 40659_2016_95_MOESM11_ESM.docx]

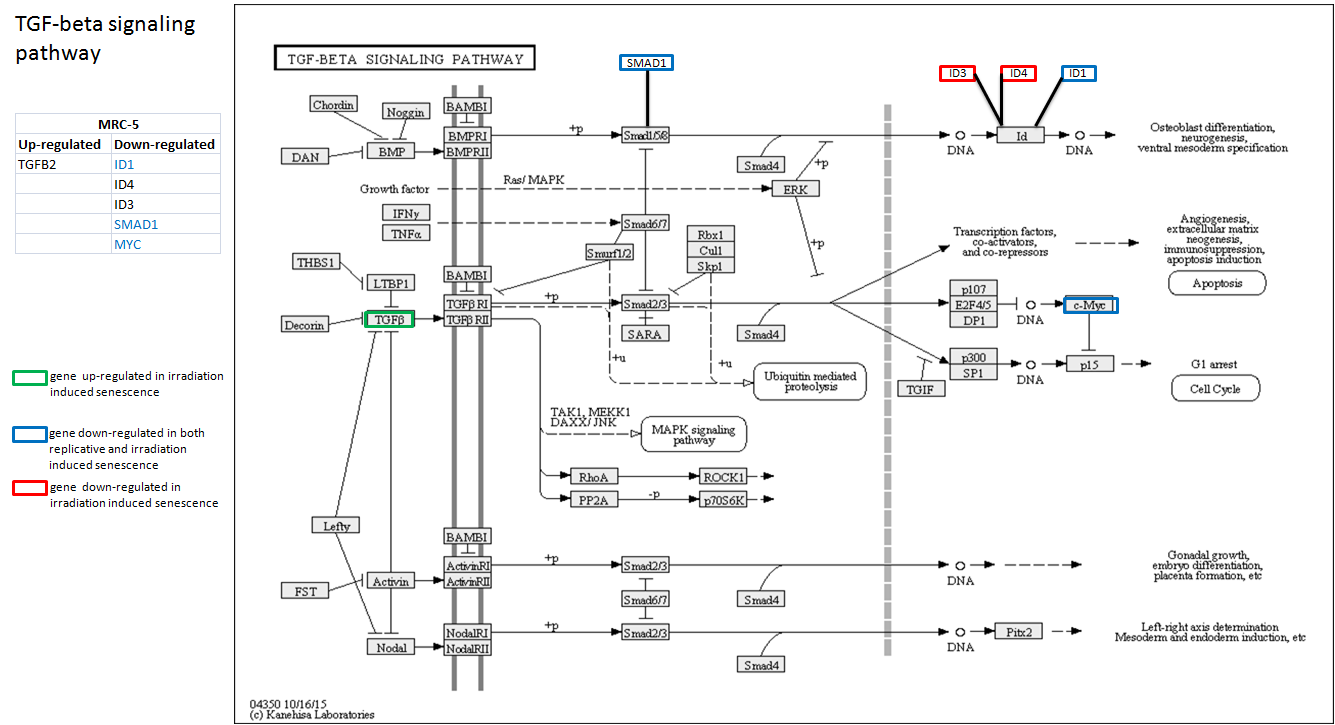

Supplement: Supplementary file 12 — 10.1186/s40659-016-0095-2 Regulation of genes of TGF-beta signaling pathway during senescence induction in MRC-5 strains. Genes of the “TGF-beta signaling” pathway which are significantly up- (green) and down- (red) regulated (log2 fold change >1) during irradiation induced senescence (120 h after 20 Gy irradiation) in MRC-5 fibroblast strains. Blue color signifies genes which are commonly down-regulated during both, irradiation induced and replicative senescence. [file 40659_2016_95_MOESM12_ESM.docx]
