# Supplementary material for: Conserved genes and pathways in primary human fibroblast strains undergoing replicative and radiation induced senescence
Source: Biol Res. 2016 Jul 28;49:34. doi: 10.1186/s40659-016-0095-2 (PMC4963952; doi:10.1186/s40659-016-0095-2)
Supplement: Supplementary file 2 [file 40659_2016_95_MOESM1_ESM.docx]

Caspase-3

   

         Bax

      

 HFF

PD16

MRC-5

 PD32

Control without irradiation

120 min after 20 Gy irradiation

                       Positive control 

(2.0mM Staurosporine treatment for 96hrs)

Control without irradiation

120 min after 20 Gy irradiation

                       Positive control 

(2.0mM Staurosporine treatment for 96hrs)

17kDa

 21kDa

Tubulin

   

 55kDa
